# New-onset vitiligo following proprotein convertase subtilisin/kexin type 9 inhibition with evolocumab: A possible immune-mediated adverse effect

**DOI:** 10.1016/j.jdcr.2026.02.038

**Published:** 2026-02-23

**Authors:** Carlos A. Umaña Mejia, Mariana Olvera Morales, Alan Jurado Orozco, Jose D. Burgos

**Affiliations:** a Universidad Autónoma de Guadalajara, Facultad de Medicina, Guadalajara, Jalisco, Mexico; b Internal Medicine Resident, University Medical Center of El Paso, Texas Tech University Health Sciences Center, El Paso, Texas; c Hospitalist Department, Director, University Medical Center of El Paso, Texas Tech University Health Sciences Center, Paul L. Foster School of Medicine, El Paso, Texas

**Keywords:** biologic therapy, drug-induced vitiligo, evolocumab, hyperlipidemia, immune-mediated adverse event, PCSK9 inhibitor, pharmacovigilance, skin of color, vitiligo

## Introduction

Proprotein convertase subtilisin/kexin type 9 (PCSK9) inhibitors such as evolocumab and alirocumab have become an important therapeutic option for patients with hypercholesterolemia who require additional low-density lipoprotein cholesterol lowering beyond traditional treatments such as statins and ezetimibe. Their efficacy and safety for primary and secondary prevention of atherosclerotic cardiovascular disease have been well established in both randomized controlled trials and meta-analyses.^1^^,^^2^

In general, PCSK9 inhibitors have been well tolerated by most patient populations, but pharmacovigilance analyses have revealed a broad spectrum of adverse events related to their use. The most common side effects reported are flu-like symptoms and mild neurocognitive complaints. The most common dermatologic adverse events related to the use of PCSK9 inhibitors are injection-site pain, erythema, bruising, pruritus, and rash.^3^^,^^4^

Vitiligo is an acquired depigmenting disorder characterized by the presence of well-demarcated white patches of varying sizes on the skin. It is considered a multifactorial disease involving genetic, environmental, and immunological factors. According to the study by Gupta et al, most cases occur between ages 30 and 70 years.^5^ In a recently published analysis of the Food and Drug Administration Adverse Event Reporting System, drug-induced vitiligo was predominantly observed with the use of monoclonal antibodies, most specifically immune checkpoint inhibitors such as pembrolizumab and nivolumab. Only 3.4% of reported cases occurred in patients aged 70 to 80 years, making new-onset vitiligo in this age group relatively uncommon.^6^ To our knowledge, no prior case reports have linked PCSK9 inhibitors with new-onset vitiligo. We describe the case of a 77-year-old male who developed new-onset vitiligo 2 months after being started on evolocumab to treat hyperlipidemia, with no identifiable risk factors for the disease.

## Case report

A 77-year-old male with a history of coronary artery disease, coronary artery bypass grafting 20 years prior, and percutaneous coronary intervention with 3 stents placed 7 years ago presented with progressive depigmented skin lesions. His long-term medications included aspirin, clopidogrel, atorvastatin, and metoprolol. Evolocumab was initiated by his cardiologist for additional low-density lipoprotein cholesterol reduction 52 days before the onset of symptoms.

The patient reported that approximately 2 months after starting evolocumab, his son noticed a depigmented macule on his back, which had not been present previously. Later that same day, he observed the appearance of new depigmented macules on his hands. The lesions gradually increased in number over the following weeks.

He was evaluated by a dermatologist, who clinically diagnosed nonsegmental vitiligo based on the characteristic distribution and morphology of the lesions; no skin biopsy was performed. Treatment was initiated with topical ruxolitinib 1.5% cream twice daily and narrow-band ultraviolet B phototherapy twice weekly.

Despite adherence to treatment, the patient developed progressive depigmentation involving most of his body surface, consistent with generalized vitiligo. With ongoing therapy, the disease has shown partial control, but new lesions continue to appear intermittently, and repigmentation has not been achieved.

**Fig 1:**
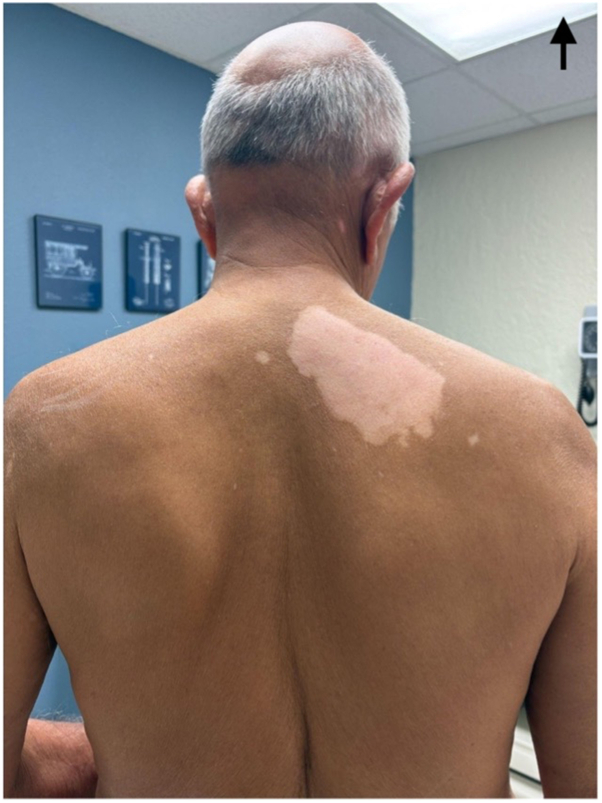
Vitiligo original depigmentation spot on back of patient.

**Fig 2:**
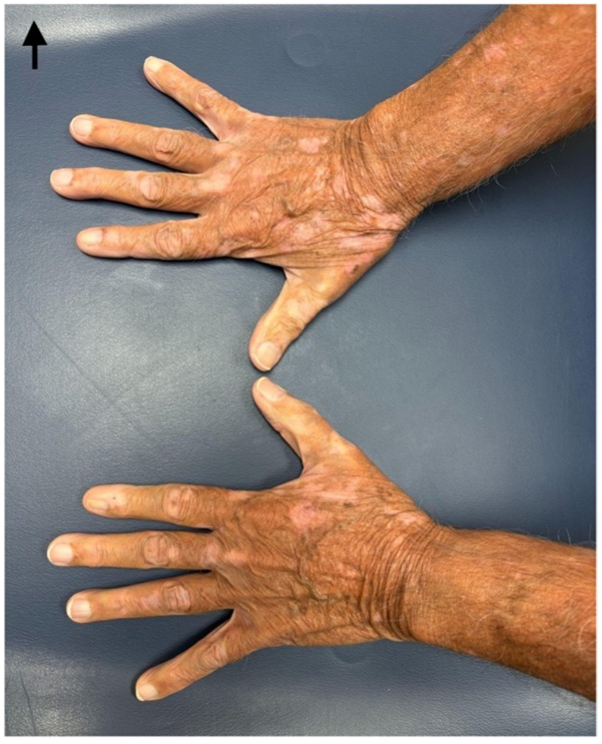
Vitiligo on patient’s hands.

**Fig 3:**
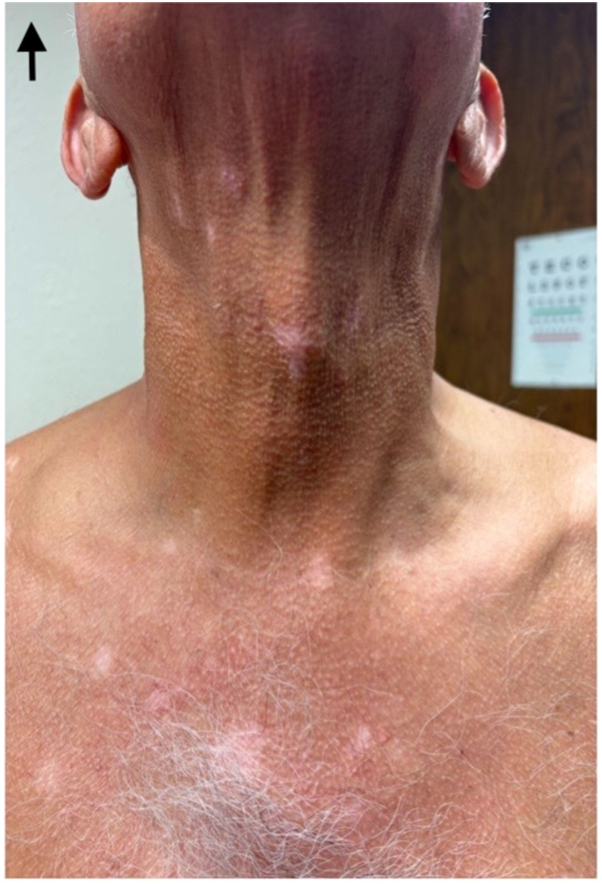
Vitiligo on patient’s chest and neck.

**Fig 4:**
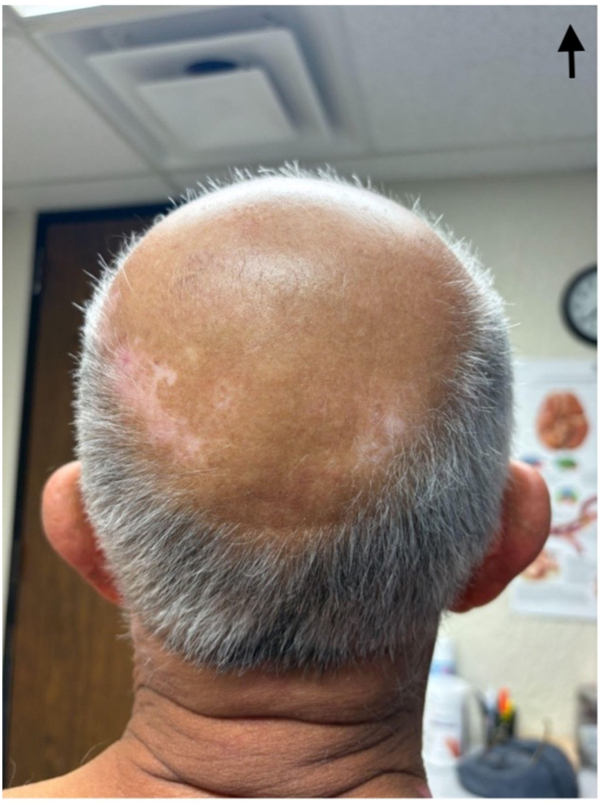
Vitiligo on patient’s head.

Importantly, the patient continues evolocumab therapy, as his dermatologist and cardiologist advised that vitiligo is not a recognized adverse effect of the medication. He denies any personal or family history of autoimmune disease, including thyroid disease, as well as any prior dermatologic conditions. Given the temporal association between evolocumab initiation and depigmentation onset, a potential drug-induced mechanism was considered, although causality remains uncertain. Current status of the lesions is shown in Fig 1, Fig 2, Fig 3, Fig 4.

## Discussion

The temporal relationship between the start of evolocumab therapy and the onset of vitiligo, plus the absence of any major risk factors, raises the question about a possible drug-induced reaction, although causality is not certain. Vitiligo is not reported as an adverse event in drug adverse event reporting systems. Interestingly, a recent Mendelian randomization analysis suggested that PCSK9 inhibitors might offer a protective effect against the development of vitiligo, implying that at greater scale and population level, PCSK9 inhibitors are unlikely to induce depigmentation disorders.^7^ However, an idiosyncratic immune reaction in susceptible individuals cannot be excluded.

Although PCSK9 inhibitors are primarily used as lipid-lowering therapies, they may also exert a broader immunomodulatory effect. PCSK9 inhibition increases Major Histocompatibility Complex class I expression and enhances CD8+ T-cell responses, providing a plausible mechanism for melanocyte autoimmunity. These pleiotropic effects, including modulation of oxidative stress and inflammatory pathways, could alter immune homeostasis in predisposed patients and potentially trigger cutaneous immune manifestations such as vitiligo.^8^^,^^9^

Emerging translational studies in human tissues and early clinical trials have begun to explore these immune effects, suggesting that PCSK9 inhibition may enhance Major Histocompatibility Complex class I expression and CD8+ T-cell infiltration in certain tumor settings, although systemic immune consequences in nononcologic patients remain unclear.^10^ The Naranjo adverse drug reaction probability score in this case was 3, indicating a possible relationship between evolocumab and the observed vitiligo. The absence of a biopsy is 1 of the limitations for our report, but clinical presentation and response to treatment deemed a biopsy unnecessary for diagnosis.

This case represents, to our knowledge, the potential first case of vitiligo associated with the use of evolocumab. Although current evidence does not support a connection between the use of PCSK9 inhibitors and the development of vitiligo, the temporal relationship and biological plausibility warrant further investigation. Notably, other biologic agents have been reported to induce drug-related vitiligo, suggesting that similar immune-mediated mechanisms could be involved in this case. Physicians should be aware of possible cutaneous reactions that may present with the use of PCSK9 inhibitors. Given the increasing and widespread use of PCSK9 inhibitors such as evolocumab in contemporary cardiovascular practice, particularly among patients with established atherosclerotic cardiovascular disease and statin intolerance, the absence of prior reports of vitiligo may suggest that this represents a rare or under-recognized adverse event rather than a common drug-related phenomenon. Mild or delayed clinical manifestations may go by unnoticed or unreported. Additional case reports and larger observational studies are needed to determine whether a causal relationship exists between PCSK9 inhibitors and the development vitiligo.

## Conflicts of interest

None disclosed.
